# Exploring the complexities of disproportionality analysis in pharmacovigilance: reflections on the READUS-PV guideline and a call to action

**DOI:** 10.3389/fphar.2025.1573353

**Published:** 2025-04-09

**Authors:** Tarek A. Hammad, Melissa Naylor, Dona M. Ely, Simon Davies

**Affiliations:** ^1^ Medical Safety of Marketed Products Development and Plasma-Derived Therapies, Patient Safety and Pharmacovigilance, Takeda Development Center Americas, Inc., Cambridge, MA, United States; ^2^ R&D Data and Quantitative Sciences, Takeda Development Center Americas, Inc., Cambridge, MA, United States; ^3^ Signal Management and Innovation, Patient Safety and Pharmacovigilance, Takeda Development Center Americas, Inc., Cambridge, MA, United States

**Keywords:** disproportionality analysis, pharmacovigilance, signal detection, spontaneous reporting systems, drug safety evaluation, READUS-PV guideline

## Abstract

The use of disproportionality analysis (DA) in pharmacovigilance to detect signals of disproportionate reporting (SDRs) has gained popularity, resulting in a surge of publications based on aggregate analysis of spontaneously reported adverse events (AE). The recently published READUS-PV guideline, designed to standardize reporting practices of DA-based publications, is a commendable first step toward standardizing DA reporting; however, it will not overcome totally many of the inherent limitations of DA including their inability to eliminate unnecessary noise in order to identify true signals. The limitations arise from the data sources of AEs, the analytic approaches, and the interpretability of the results. This article discusses those limitations, highlights the challenges posed by the premature publication of safety signals derived from spontaneous reports, and evaluates the READUS-PV guideline’s potential to improve interpretation of DA results. The article emphasizes that effective reporting of safety signals is only the first step; a broader, coordinated effort is necessary to establish clear scientific boundaries on what aspects of signal detection should be publicly shared to prevent unwarranted alarm and misinterpretation. It proposes the formation of a consortium, or a similar effort, led by regulators and involving academia and industry, to develop standards for the responsible validation and sharing of safety signal data.

## 1 Introduction

CIOMS VIII^1^ defines a safety signal as “Information that arises from one or multiple sources (including observations and experiments), which suggests a new potentially causal association, or a new aspect of a known association, between an intervention and an event or set of related events, either adverse or beneficial, that is judged to be of sufficient likelihood to justify verificatory action.” The notion of a drug safety signal has existed for a long time. However, with the approval of more medications each year and a growing number of individuals using them, the volume of adverse event reports submitted to both manufacturers and regulatory agencies has steadily increased. In some instances, pharmacovigilance data may serve as the primary or sole source of safety information, particularly for rare adverse drug reactions not detected in pre-market clinical trials. While such cases highlight the value of spontaneous reporting, they also reinforce the need for rigorous assessment to minimize the risk of overinterpretation. The field of pharmacovigilance has seen a significant increase in publications using disproportionality analysis (DA) to identify potential safety signals from the reported adverse events (Sartori et al., 2023). These publications utilize spontaneous reporting systems such as FAERS and VigiBase and employ statistical metrics to flag signals of disproportionate reporting (SDRs). This increase can be attributed to the growing availability of public access to these data sources. These databases provide researchers with large repositories of spontaneous adverse event reports, enabling independent investigations into potential drug safety issues without requiring direct involvement from regulatory agencies or pharmaceutical companies.

However, many DA-based publications overstate the significance of their findings (Mouffak et al., 2021), often overlooking the inherent biases in spontaneous reporting data and the rigorous evaluation required to validate signals. They tend to focus solely on differences in relative reporting without considering the patient population, the background disease, the concomitant use of drugs, the concurrent medical conditions of the patients, or the nature of the adverse event itself, which are critical aspects for contextualizing safety signals (Hammad et al., 2023). A study that assessed 100 disproportionality analysis (DA) studies highlighted significant heterogeneity in methodologies and reporting practices, raising concerns about the reliability of DA-generated signals. The study found substantial variability in study populations, signal detection methods, adjustment strategies, and threshold definitions, often without justification or pre-specification in study protocols. Notably, 78% of studies lacked clear definitions for case selection, adverse drug reactions, or comparators, and 32% did not specify signal detection thresholds. These inconsistencies underscore the risk of generating misleading results and unwarranted safety concerns. The authors emphasize the need for greater methodological transparency, including pre-specified protocols, clearly defined comparators, and sensitivity analyses, to improve the reliability and interpretability of DA findings (Khouri et al., 2021). Another recent study by Bernardeau et al. (2025) critically examined the language used in pharmacovigilance publications that employ disproportionality analysis (DA) based on spontaneously reported AEs. The study found that many of these publications make causal statements that may not be fully supported by the data, potentially leading to exaggerated perceptions of drug safety risks. This highlights the necessity for cautious interpretation and reporting of DA findings to prevent misrepresentation of drug safety profiles. This trend has raised concerns among regulators, drug safety researchers, and journal editors, as the proliferation of such studies risks propagating unverified safety signals, potentially influencing patients and clinical practice.

## 2 Limitations of disproportionality analysis in drug safety research

While DA methods using the spontaneously reported AEs are valuable for hypothesis generation, they are inherently limited in their ability to eliminate unnecessary noise and identify true signals. The limitations arise from the data sources, the analytic approaches, and the interpretability of the results.

### 2.1 Limitations of the spontaneously reported AE data

The limitations of the information in the spontaneous reporting safety databases are well known and may include both underreporting and overreporting. Many AEs go unreported, while others may be reported multiple times by patients, healthcare professionals, and drug manufacturers, leading to duplication within the safety dataset.

Reporting rates can also be significantly influenced by external factors such as mainstream media and social media coverage, patient support programs, regulatory actions, legal cases, and public awareness. These influences introduce bias into the data source and complicate the interpretation of SDRs and DA.

Furthermore, The quality and completeness of reports can vary widely, with many lacking the information needed for meaningful evaluation. AEs in the safety databases often lack critical details on dosage, duration of drug exposure, concurrent medications, comorbidities, and lifestyle factors. These missing data points are key for accurately assessing the risk of AEs but are challenging to account for, further limiting the reliability of the conclusions derived from analyses of these datasets.

Additionally, spontaneous reports do not contain reliable denominator data (i.e., the number of patients exposed to the drug), which prevents estimation of incidence rates or comparative risk.

### 2.2 Methodological challenges of disproportionality analysis metrics

Disproportionality analysis (DA) employs various statistical metrics to identify SDRs. Some of the commonly used metrics include Information Component (IC), Multi-item Gamma-Poisson-Shrinker (MGPS), Reporting Odds Ratio (ROR) and Proportional Reporting Ratio (PRR) (Cutroneo et al., 2024)^2^.

The IC metric uses a Bayesian framework to adjust for sparse data by incorporating prior probabilities based on background reporting rates. While this approach mitigates random noise, the IC025 values (the lower bound of the 95% confidence interval) may not always provide clear insights into the strength or clinical relevance of an association. The Empirical Bayes Geometric Mean (EBGM) shares some of the attributes of the IC, as it also relies on Bayesian principles to stabilize disproportionality estimates.

The ROR, by contrast, is highly sensitive to rare events and small sample sizes, where even a few additional reports can disproportionately inflate values, resulting in false-positive findings. Furthermore, the ROR assumes uniform reporting practices across all drugs and events, an assumption that is rarely valid given the variability in monitoring intensity and regulatory requirements. Additionally, the need for arbitrary adjustments (e.g., adding a continuity correction of 0.5 to all cells in the 2 × 2 table) to calculate ROR in the absence of cases among comparator drugs can further introduce bias and artificially inflate associations.

Similarly, the Proportional Reporting Ratio (PRR), while simple to calculate, might overestimate disproportionality for frequently reported AEs, making it less reliable in cases of common drug-event combinations.

These examples illustrate some of the methodological limitations inherent in DA metrics and underscore the need for careful interpretation of their results, taking into account clinical context. Overall, these limitations highlight the importance of contextualizing DA findings within the broader clinical and regulatory landscape. While these metrics are useful tools for hypothesis generation as already mentioned, they should not be used in isolation to draw definitive conclusions about drug safety (Fusaroli et al., 2024a).

### 2.3 Complexities of the interpretation of disproportionality analysis results

Interpretation of DA results is limited by the potential for erroneous conclusions due to 1) the increased chance of declaring statistical significance when analyzing large samples or making multiple comparisons, and 2) many potential sources of bias. DA conducted on large safety datasets can yield erroneous conclusions that appear statistically significant due to the high volume of data and the number of comparisons being made. The findings may be a result of random variation rather than a true signal. In addition, researchers often fail to account for confounders. Many drugs are widely prescribed for a range of conditions, often to individuals with underlying comorbidities or advanced age. These factors might independently increase the risk of a reported AE but may be unaccounted for in the analysis, often because they are missing in the data source. As a result, SDRs have low yield in identifying clinically actionable safety findings when presented in isolation (Loke et al., 2024), without the broader context of a full signal evaluation (Hammad et al., 2023).

A notable instance of a false-positive signal involves the association between statins and amyotrophic lateral sclerosis (ALS). Early DA indicated a disproportionate number of ALS reports among statin users, raising concerns about a potential link (Edwards et al., 2007). However, FDA investigation (Colman et al., 2008) as well as subsequent epidemiological studies (Chang et al., 2021), found no increased risk of ALS with statin use, demonstrating that the initial signal was false.

Another example of a disproportionality analysis (DA) yielding a false-positive signal involves the association between sodium–glucose cotransporter-2 (SGLT2) inhibitors and acute kidney injury (AKI). Initial pharmacovigilance studies utilizing DA suggested an increased risk of AKI among users of SGLT2 inhibitors (Perlman et al., 2017). However, incorporating active-comparator design in DA, there was no safety signal for AKI, in effect the approach enhanced the robustness of signal detection by reducing the effect of confounding and channeling bias (Alkabbani and Gamble, 2023). Additionally, subsequent robust studies collectively demonstrate that the initial safety signal suggesting an association between SGLT2 inhibitors and AKI was not supported. On the contrary, SGLT2 inhibitors may offer a protective effect against AKI (Menne et al., 2019; Pasternak et al., 2020; Alkabbani et al., 2021; Zhuo et al., 2022). More specifically, disproportionality analysis estimators are subject to biases that can impact drug safety signal detection. Key biases include signal leakage, confounding by indication, and competition bias (masking). Signal leakage occurs when a drug-event combination is mistakenly flagged as a safety signal, even though the AE is actually caused by a co-administered drug. Confounding by indication arises when the AE is linked to the underlying disease being treated or a related comorbidity rather than the drug itself. Competition bias happens when newer drugs with similar indications or mechanisms, and a stronger association to the AE, mask signals for the older drugs. These biases highlight the complexities in interpreting disproportionality analysis (Gravel and Douros, 2023).

That said, several methodological approaches have been proposed to mitigate some of the biases that might be encountered in disproportionality analysis. For example, methods such as stratification by therapeutic area, use of comparator drug-event pairs, and sensitivity analyses have been suggested to minimize the influence of confounding by indication and competition bias (Alkabbani and Gamble, 2023; Arnaud et al., 2016; Grundmark et al., 2014). Incorporating such adjustments may help reduce the risk of false-positive safety signals and improve the interpretability of DA findings.

Subgroup analyses, such as gender-based risk differences, have become increasingly common in DA publications, yet they present significant methodological challenges and risks of misinterpretation. The data in spontaneous reporting systems does not support such nuanced investigations, as they lack structured demographic and clinical data necessary for appropriate stratification. In these databases, apparent gender differences in AE reports can easily result from various reporting biases, including differences in healthcare-seeking behavior, gender-specific disease incidence, prescribing practices, or coincidental overrepresentation. Without careful consideration of these factors, DA-based subgroup analyses risk misleading readers and could contribute to unwarranted safety concerns or misinformed clinical decisions. It is critical to caution against overreliance on subgroup-based findings and advocate for a more responsible approach to DA-based research.

While we acknowledge that subgroup analyses have been proposed as part of routine first-pass signal detection, as recommended in the IMI PROTECT Good Signal Detection Practices (Wisniewski et al., 2016), their application carries inherent risks of confounding and overinterpretation. The study by Seabroke et al. (2016) illustrates the potential utility of subgroup analyses but also highlights significant methodological limitations. The reliance on disproportionality metrics without adequately accounting for confounders—such as differential drug utilization patterns, comorbidities, and reporting biases—raises concerns about the validity of subgroup-specific signals. Moreover, the increased granularity of these analyses amplifies the risk of statistical noise, as smaller sample sizes within subgroups can yield unstable disproportionality estimates, potentially exaggerating or masking true signals. Given these concerns, while subgroup analyses may serve as an exploratory tool, their results should be interpreted with extreme caution. Without subsequent validation through robust studies capable of adjusting for confounders and assessing true risk estimates, subgroup-based DA findings from spontaneous reporting databases remain speculative and should not be used as standalone evidence for regulatory or clinical decision-making.

Given all these limitations with current disproportionality statistics, significant improvements in signal detection may require new methodologies that diverge from existing approaches (Wisniewski et al., 2016). While various disproportionality statistical metrics are available, they are more or less conceptually similar (Wisniewski et al., 2016). A study by Candore et al. (2015) compared the performance of several signal detection algorithms across multiple spontaneous reporting databases from national agencies, international pharmacovigilance organizations, and pharmaceutical companies. The results demonstrated significant variability in performance across databases, with increases in sensitivity often accompanied by decreases in precision. No single algorithm consistently outperformed others in flagging true signals, and performance depended heavily on database-specific characteristics and thresholds applied to statistical signals. Over a drug’s lifecycle, signal detection algorithms exhibited a decline in precision due to several factors. The increasing volume and diversity of spontaneous reports dilute the signal-to-noise ratio, making it harder to distinguish true safety signals from irrelevant associations. Broader patient exposure, including off-label use and diverse populations, further contributes to this complexity, as does the accumulation of background reports and evolving reporting and competition biases mentioned earlier influenced by media, regulatory actions, and public awareness and other drugs, respectively. This underscores the need for continual refinement of signal detection methodologies and to evaluate methods within the specific context of a given database and the lifecycle stage of the drug.

A recent study found significant variability in the methodologies used to assess the validity of disproportionality signals, reflecting a lack of standardization in post-detection signal evaluation (Fusaroli et al., 2023). Given this heterogeneity, the establishment of harmonized best practices for post-detection validation would enhance the consistency and credibility of pharmacovigilance assessments. To address these caveats, regulatory reviewers and pharmaceutical companies apply investigative methods to ensure a comprehensive assessment of safety signals, which is essential for confirming the validity and relevance of DA findings. Regulatory reviewers, with their technical expertise, approach disproportionality analysis (DA) with a deeper understanding of its limitations and integrate it into a broader evaluation process. This thorough review involves in-depth examination of biological plausibility, safety data from other drugs in the same class, information from available clinical trials and pharmacoepidemiologic studies, preclinical data, and the application of causality frameworks tailored for drug safety (e.g., Hammad et al., 2023) to substantiate and clarify any causal relationships. In contrast, members of the general public or researchers who rely solely on the numerical output of SDR metrics without this context, no matter how well-reported, can inadvertently contribute to false positives or over-identification of safety concerns. This distinction underscores the critical role of expertise and comprehensive approaches in ensuring the reliability of signal detection and evaluation in pharmacovigilance. Unfortunately, many published manuscripts fail to incorporate these steps, leading to premature, potentially inaccurate, conclusions.

## 3 READUS-PV guideline

The recently published paper, “The Reporting of Disproportionality Analysis in Pharmacovigilance: Spotlight on the READUS-PV Guideline,” highlights the newly developed guideline for reporting DA analyses (Fusaroli et al., 2024a). The authors endorse the READUS-PV guideline as the first standardized framework for reporting DAs based on spontaneously reported AEs. The paper begins with a comprehensive background of DA with its many challenges and goes on to present an overview of the READUS-PV guideline, which was published elsewhere in detail (Fusaroli et al., 2024b; Fusaroli et al., 2024c). The guideline was developed using a structured methodology that involved a modified Delphi process and incorporated feedback from pharmacovigilance experts across academia, industry, and regulatory bodies.

The READUS-PV guideline is intended to address the ever-increasing challenge of the reporting of DAs without a context in pharmacovigilance. By establishing a checklist for DA reporting, the guideline aims to improve transparency and reproducibility. Optimally, its potential impact would be greatly enhanced by empirical validation through real-world application, as its practical value in reducing false positives and improving the utility of DA findings, despite being developed through a robust consensus process, remains speculative. Demonstrating its effectiveness through case studies or retrospective analyses of previously published DA findings could have substantiated its claims and provided a stronger foundation for its adoption. Nonetheless, while the READUS-PV guideline has not undergone retrospective validation, it aligns with the standard approach for reporting guidelines, where prospective validation through real-world implementation and user feedback is considered more informative. Future research should assess its adoption, usability, and overall impact on reporting completeness to determine its effectiveness in improving pharmacovigilance practices.

While the intention to elevate the rigor of DA reporting is greatly appreciated, the framing of DA as a “study design” in discussions of the READUS-PV guideline, as well as in some other publications, is particularly concerning and warrants careful scrutiny. While DA lacks key elements of traditional study designs, such as predefined exposure definitions, confounder control, and statistical power calculations, some researchers consider it a structured approach within the broader category of signal detection methodologies, similar to symmetry sequence analyses or TreeScan. We believe DA remains primarily a hypothesis-generating tool rather than an analytical framework designed to estimate risk. Unlike robust study designs, such as clinical trials or cohort studies, DA lacks the elements of scientific rigor needed to contribute in establishing causality. Moreover, DA in pharmacovigilance does not typically involve true “non-cases” or “or “comparator” in the manner of traditionally designed studies. Thus, introducing terms like “non-cases” or “comparator” in DA implies a structured control that does not exist. In DA, statistical methods calculate the relative reporting frequency of a drug-event pair by comparing it with all other drugs for the same event or with all other events for the same drug within the dataset. This approach fundamentally differs from study designs with a clearly delineated comparator group. Referring to DA as a study design risks overstating its scientific validity and could mislead less-experienced readers or practitioners into equating DA findings with causal relationships. The primary purpose of DA, as established in the literature and regulatory guidance, is to flag safety signals for further investigation. DA is an analytics tool, and its findings should at most represent a flag for the need of further assessment. Positioning DA appropriately within the field of pharmacovigilance will ensure that its role as an initial hypothesis-generating screening tool is neither overstated nor misinterpreted as providing the scientific rigor of a formal study design.

In practice, as already mentioned, regulators and drug manufacturers conduct rigorous, multi-faceted assessments before sharing safety signal findings with patients and healthcare professionals (Hammad et al., 2023). It is worth noting that the READUS-PV explanation and elaboration paper (Fusaroli et al., 2024c) includes explicit recommendations for contextualizing DA findings within existing literature and other consulted sources, including reference to guidelines for causality assessment. While the guideline acknowledges the importance of contextual evidence, an explicit mandate for integrating diverse sources of data into signal validation and confirmation would help ensure that DA findings are interpreted within a robust and scientifically sound framework.

The success of the READUS-PV guideline will depend on iterative refinement based on feedback from its real-world application. Establishing a mechanism for stakeholders—regulators, researchers, and industry professionals—to provide input on their experiences with the guideline would help identify areas for improvement and ensure its long-term relevance. Encouraging dialogue and collaboration across the pharmacovigilance community will be key to enhancing the quality and impact of DA-based publications. By incorporating these recommendations, the pharmacovigilance community can build upon the READUS-PV guideline to advance not only the transparency of DA reporting but also the overall reliability and utility of disproportionality analysis in drug safety research.

## 4 Commentary and call to action

We established that premature publication of unvalidated SDRs carries significant risks including creating unnecessary alarm among healthcare providers and regulators and confusion among readers unfamiliar with the limitations of post-marketing spontaneous reports and disproportionality analysis. Without proper regulatory, clinical, and methodological context, these findings might be misinterpreted as definitive evidence of a drug’s adverse effects, rather than preliminary signals requiring further investigation. Therefore, it might be prudent for scientific journals to refrain from publishing manuscripts relying solely on DA findings without the attendant context. The proliferation of such studies could overwhelm the field, diluting the scientific literature with findings that lack robustness and utility.

It is important to clarify that the READUS-PV guideline does not aim to provide recommendations for good practices in signal detection or strategies for bias minimization in DA. Instead, it focuses on standardizing reporting practices. The READUS-PV guideline represents an important step toward increasing scientific rigor in DA reporting by emphasizing transparency in case definitions, reference group selection, and bias handling strategies. As such, it should be viewed in conjunction with complementary initiatives, such as IMI PROTECT, which provide broader guidance on methodological improvements in pharmacovigilance to address the limitations inherent in DA itself.

Nonetheless, DA, again no matter how well-reported, will remain susceptible to producing misleading associations. As a field, we must recognize that providing guidance for reporting of safety signals is only a starting point; a broader, more coordinated effort is essential to establish clear scientific boundaries on what aspects of signal detection activities should be publicly shared. Without such boundaries, there is a risk of alarming patients and healthcare providers with unvalidated safety signals, potentially deterring the use of beneficial drugs. To address this, we propose to form a consortium, or to use existing appropriate scientific body, e.g., the READUS-PV working committee or a subcommittee sponsored by the International Society of Pharmacovigilance, involving key stakeholders—including academia, industry, and regulators—to set standards and best practices for sharing safety signal data responsibly. Regulators, in particular, should play a central role in this effort, ensuring that these standards are aligned with public health priorities and regulatory frameworks.

This initiative is urgent, as the current trend of leveraging safety databases is only the beginning. The future will see increased use of diverse data sources, such as social media, electronic medical records, and other real-world evidence platforms, for pharmacovigilance purposes. While these data sources hold promise, they also bring significant challenges in terms of data quality, interpretation, and potential for generating false alarms. Without disciplined, organized approaches to investigating safety signals and performing comprehensive assessments to confirm risks, the field risks becoming overwhelmed by unreliable or premature findings. A structured and collaborative approach is needed to ensure that signal detection activities advance in a scientifically rigorous and socially responsible manner.

## Data Availability

The original contributions presented in the study are included in the article/supplementary material, further inquiries can be directed to the corresponding author.

## Footnotes

1
https://cioms.ch/sd7fdh93gewd882ds/uijkwqd230sdidjs-yellow/SECURED-CIOMS-Report-WG-VIII-2010.pdf

2
https://cioms.ch/wp-content/uploads/2018/03/WG8-Signal-Detection.pdf#:∼:text=First%2C%20the%20development%20of%20statistical%20and%20analytical,signal%20detection%2C%20not%20provide%20evidence%20of%20causality
